# Supplementary material for: Cardioprotection and Suppression of Fibrosis by Diverse Cancer and Non-Cancer Cell Lines in a Murine Model of Duchenne Muscular Dystrophy
Source: Int J Mol Sci. 2024 Apr 12;25(8):4273. doi: 10.3390/ijms25084273 (PMC11050177; doi:10.3390/ijms25084273)
Supplement: Supplementary file 1 [file ijms-25-04273-s001.zip › ijms-2944531-supplementary.pdf]

Supplementary figures

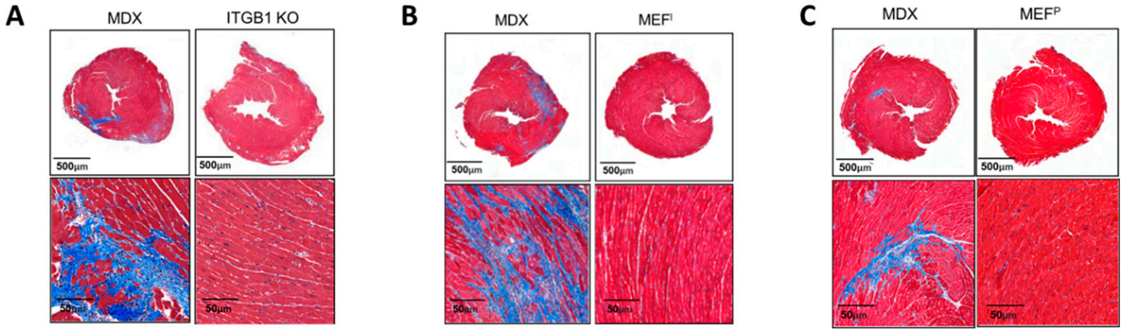

Supplementary figure S1. Injection of less aggressive PyMT ITGB1 KO cells and non-cancerous MEF^{I} and MEF^{P} cells led to a reduction in fibrosis in the hearts of MDX mice. Representative image of the heart of control MDX (left), MDX injected mice (right) with (**A**) PyMT ITGB1 KO, (**B**) MEF^{I}, and (**C**) MEF^{P} cells, stained for Masson's trichrome. Scale bar: 500 µm (top) and 50 µm (bottom).

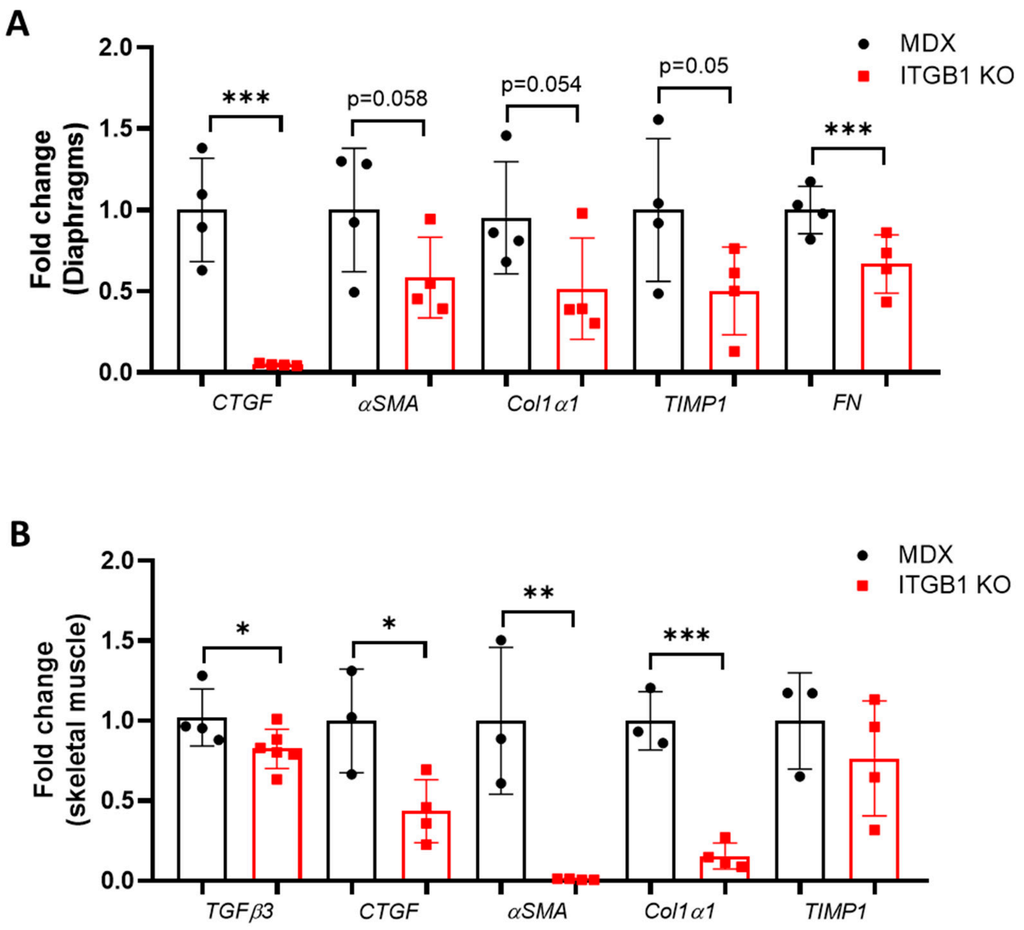

Supplementary figure S2. Injection of less aggressive PyMT ITGB1 KO cells resulted in a significant decrease in fibrotic hallmark genes of the diaphragm and skeletal muscles of MDX mice. (**A-B**) Transcription levels of fibrosis hallmark gene markers TGFβ3, CTGF, αSMA, Col1α1, and TIMP1 in of ITGB1 KO-bearing MDX mice compared to control MDX mice in the diaphragm muscles(**A**) and in the skeletal muscles (**B**), measured using qRT-PCR normalized to mB2M and βACTIN gene, respectively. Data are presented as mean ± SE. One-way ANOVA followed by Tukey post-test; (**A,B**). * $p < 0.05$; ** $p < 0.01$; *** $p < 0.001$. Each dot represents one mouse.

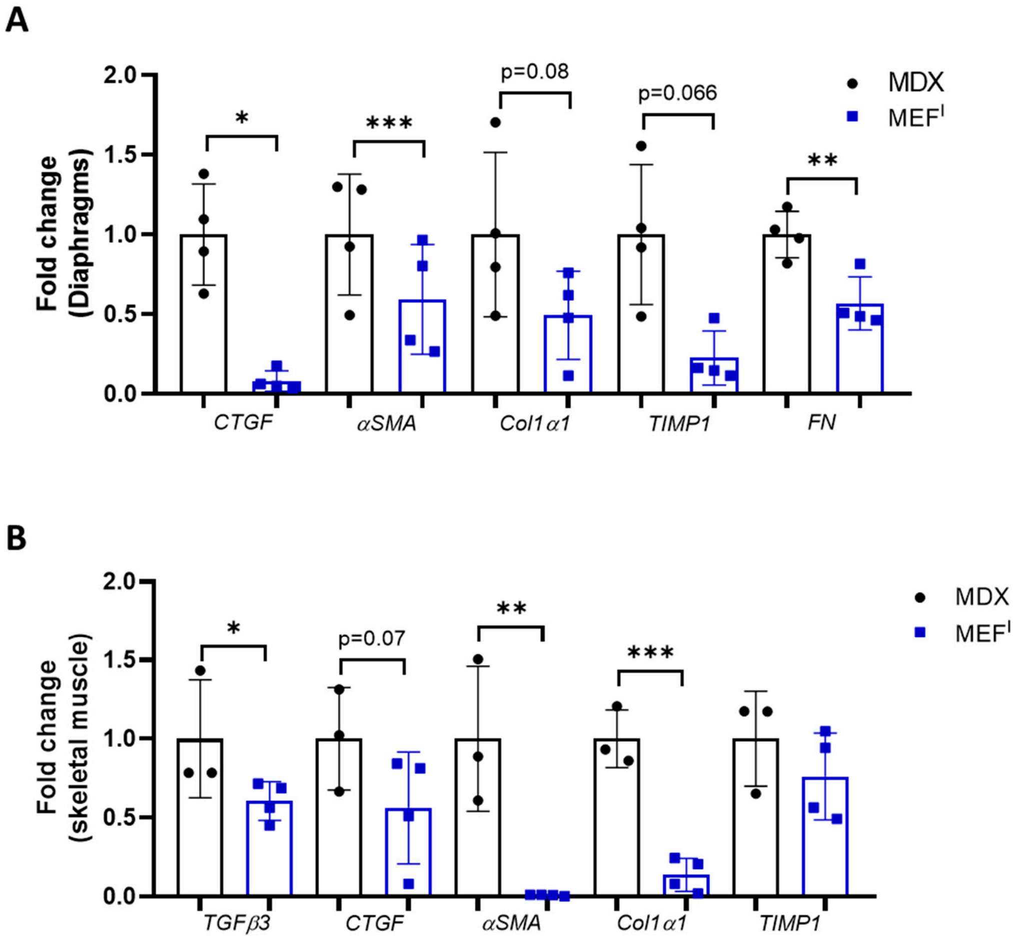

Supplementary figure S3. Injection of non-cancerous MEF^{I} cells led to a significant decrease in fibrotic hallmark genes of the diaphragm and skeletal muscles of MDX mice. (**A-B**) Transcription levels of fibrosis hallmark gene markers TGFβ3, CTGF, αSMA, Col1α1 and TIMP1 in of MEF^{I} injected MDX mice compared to control MDX mice in the diaphragm muscles(**A**) and in the skeletal muscles (**B**), measured using qRT-PCR normalized to mB2M and βACTIN gene, respectively. Data are presented as mean ± SE. One-way ANOVA followed by Tukey post-test; (**A,B**). * $p < 0.05$; ** $p < 0.01$; *** $p < 0.001$. Each dot represents one mouse.

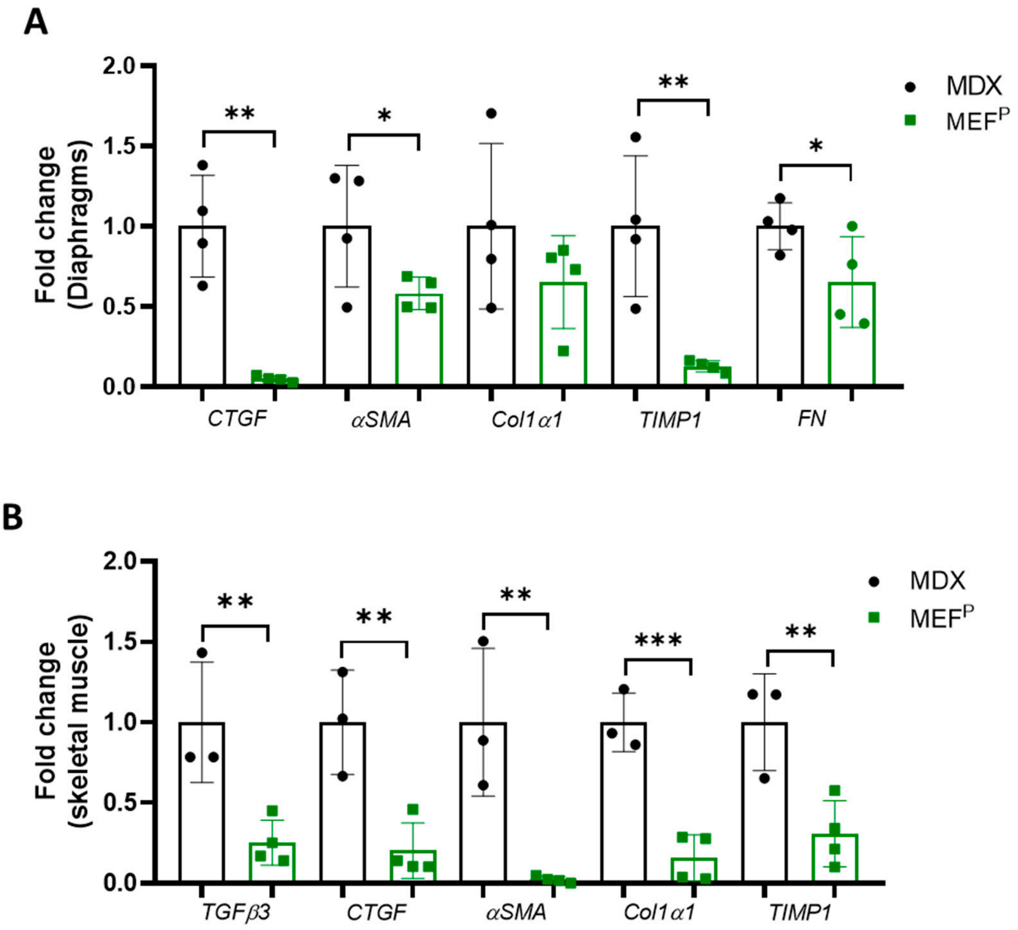

Supplementary figure S4. Injection of non-cancerous MEF^P cells led to a significant decrease in fibrotic hallmark genes of the diaphragm and skeletal muscles of MDX mice. (**A-B**) Transcription levels of fibrosis hallmark gene markers TGFβ3, CTGF, αSMA, Col1α1 and TIMP1 in of MEF^P injected MDX mice compared to control MDX mice in the diaphragm muscles(**A**) and in the skeletal muscles (**B**), measured using qRT-PCR normalized to mB2M and βACTIN gene, respectively. Data are presented as mean ± SE. One-way ANOVA followed by Tukey post-test; (**A,B**). * $p < 0.05$; ** $p < 0.01$; *** $p < 0.001$. Each dot represents one mouse.

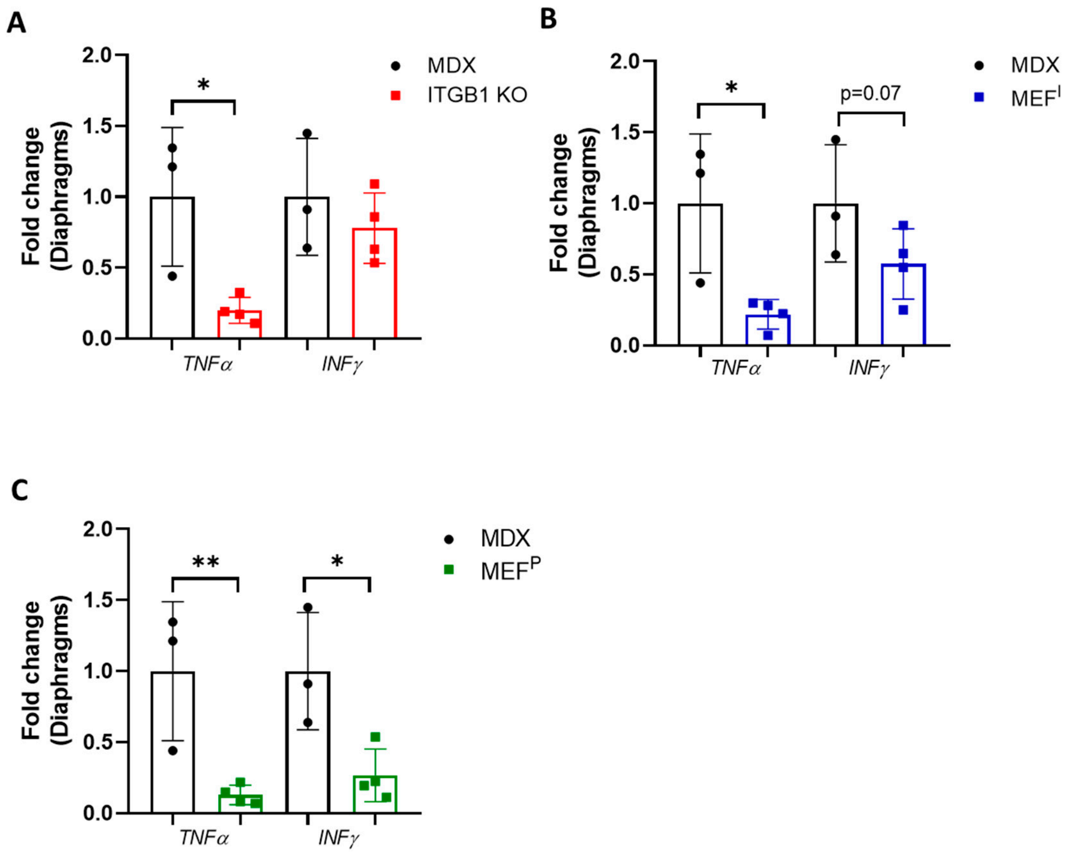

Supplementary figure S5. Injection of less aggressive PyMT ITGB1 KO cells and non-cancerous MEF^{I} and MEF^{P} cells resulted in the downregulation of the M1-polarizing hallmark gene in the diaphragm muscles of MDX mice. (**A-C**)) qRT-PCR measuring transcription levels of M1-macrophage polarizing gene: TNFα and INFγ, in the diaphragm of MDX mice (**A**) injected with ITGB1 KO, (**B**) MEF^{I} and, (**C**) MEF^{P} cells, as compared to control MDX. Measurements were obtained using qRT-PCR, normalized to housekeeping gene mB2M. Data are presented as mean ± SE. One-way ANOVA followed by Tukey post-test (**A-C**). * $p < 0.05$; ** $p < 0.01$.Each dot represents one mouse.

Gene	Forward	Reversed
Hsp90	TCGTCAGAGCTGATGATGAAGT	GCGTTTAACCCATCCAACTGAAT
mb2M	TTCTGGTGCTTGTCTCACTGA	CAGTATGTTCGGCTTCCCATTC
ACTA2	GTCCCAGACATCAGGGAGTAA	TCGGATACTTCAGCGTCAGGA
Col1α1	CTGGCGGTTCAGGTCCAAT	TTCCAGGCAATCCACGAGC
TGFβ3	CCTGGCCCTGCTGAACTTG	GACGTGGGTCATCACCGAT
CTgF	AGACCTGTGGGATGGGCAT	GCTTGGCGATTTTAGGTGTCC
TIMP	GCAACTCGGACCTGGTCATAA	CGGCCCGTGATGAGAAACT
α-SMA	GTCCCAGACATCAGGGAGTAA	TCGGATACTTCAGCGTCAGGA
FN	TGGTGGCCACTAAATACGAA	GGAGGGCTAACATTCTCCAG
POSTN	CCTGCCCTTATATGCTCTGCT	AAACATGGTCAATAGGCATCACT
G-CSF	CGTTCCCCTGGTCAGTGTC	CCGCTGGCCTGGATCTTC
GRP18	TGAAGCCCAAGGTCAAGGAGAAG	TTCATGAGGAAGGTGGTGAAGGC
Arg1	AATGAAGAGCTGGCTGGTGT	CTGGTTGTCAGGGGAGTGTT
CD163	CCTCCTCATTGTCTTCCTCCTGTG	CATCCGCCTTTGAATCCATCTCTTG
INFγ	ACAGCAAGGCGAAAAAGGATG	TGGTGGACCACTCGGATGA
TNF-α	CCCTCACACTCAGATCATCTTCT	GCTACGACGTGGGCTACAG
CCL2	GTGATGGAGGGGGTCAGGA	GGGATGGGACAGCCTAAACT
IL-1	GCAACTGTTCCTGAACTCAACT	ATCTTTTGGGGTCCGTCAACT
IL-13	AACGGCAGCATGGTATGGAGTG	TGGGTCCTGTAGATGGCATTGC

Supplemental Table S1: The sequences of the oligonucleotides used for qRT-PCR of the indicated genes.
